# Effects of dietary supplementation of polysaccharide from Agaricus blazei Murr on productive performance, egg quality, blood metabolites, intestinal morphology and microbiota of Korean quail

**DOI:** 10.5713/ab.23.0441

**Published:** 2024-04-01

**Authors:** Liang Hong, Zheng Ma, Xueyi Jing, Hua Yang, Jifei Ma, Lei Pu, Jianbin Zhang

**Affiliations:** 1 Tianjin Key Laboratory of Agricultural Animal Breeding and Healthy Husbandry, College of Animal Science and Veterinary Medicine, Tianjin Agricultural University, Tianjin 300392, China; 2 Tianjin Key Laboratory of Green Ecological Feed, Tianjin 301800, China; 3 College of Animal Science and Technology, Guangxi University, Guangxi 530000, China

**Keywords:** Agaricus blazei Polysaccharide, Egg Quality, Feed Additives, Intestinal Microorganisms, Quail

## Abstract

**Objective:**

This study aimed to investigate the effects of dietary supplementation with Agaricus blazei polysaccharide (ABP) at varying concentrations on the performance, egg quality, blood biochemistry, intestinal morphology, and microflora of quail.

**Methods:**

The study involved a total of 2,700 Korean quails, which were randomly divided into three groups. The measured variables encompassed productive performance, egg parameters, carcass parameters, serum metabolites, immune response parameters, antioxidative properties, and gut microbiome.

**Results:**

The addition of ABP did not have a significant effect on average daily feed intake. However, it was found to increase the average daily egg weight and egg production rate, reduce the feed-egg ratio. There were no significant impacts on egg quality measures such as egg shape index, egg yolk index and color, egg yolk and protein content. However, ABP supplementation significantly increased the Hough unit (p<0.01) and decreased the rate of unqualified eggs (p<0.01). Regarding serum parameters, the inclusion led to an increase in total protein concentration (p<0.05) and a reduction in low-density lipoprotein cholesterol (p<0.05). There were no significant effects observed on immune indicators such as immunoglobulin A (IgA) and IgM. ABP supplementation increased the levels of serum antioxidant indicators, including glutathione peroxidase, total superoxide dismutase (p<0.05), and total antioxidant capacity colorimeter (p<0.05). Furthermore, ABP supplementation significantly elevated the intramuscular fatty acid content in quail meat. Additionally, ABP supplementation demonstrated a significant improvement in the diversity of gut microbiota and induced alterations in the composition of the gut microbiota.

**Conclusion:**

The findings of this study indicate that dietary supplementation of ABP enhanced production performance and antioxidant capacity while increasing the levels of polyunsaturated fatty acids in quail muscle.

## INTRODUCTION

The domestic quail has recently gained significant attention in the poultry industry due to the increasing consumption of its products, such as eggs and meat. Quail is also commonly used as a laboratory animal model for scientific research due to its high resistance to avian diseases [1,2]. Quail eggs are an ideal food for brain health and development, as they are low in fat and contain more essential amino acids and minerals such as calcium, phosphorus, and iron [3]. Quail meat, particularly the leg and breast, is considered an important source of many essential nutrients, including essential amino acids, as well as various monounsaturated and polyunsaturated fatty acids (PUFA) [4].

Due to the ban on the addition of antibiotic growth promoters (AGP) to feed in the poultry industry, there is a growing need for safe alternative feed ingredients. As a result, considerable attention has been paid to the development of alternatives to AGP. For animal feed supplements, administration of mushroom polysaccharides has shown immunomodulation [5], anti-oxidation [6], anti-inflammation [7], and regulation of intestinal microbiota effects [8]. Agaricus blazei Murr (AbM) is a medicinal mushroom with great commercial potential and various health-promoting functions. AbM contains an abundance of bioactive substances, including polysaccharides, lipids (including ergosterol and sterols), proteins, vitamins B, C, and D, and phenolic compounds. Multiple studies have claimed that Agaricus blazei polysaccharide (ABP) has immunomodulatory and anti-inflammatory effects and is also believed to have curative properties for bacterial infections.

The purpose of this study was to investigate the effects of dietary supplementation of ABPs with high and low gradients on the performance, egg quality, blood metabolites, intestinal morphology, and microbiota of Korean quails.

## MATERIALS AND METHODS

### Polysaccharide used in study

Agaricus blazei polysaccharide was purchased from Shengqing Biotechnology Co. Ltd (Xi’an China), the purity was 70%.

### Animals and experimental diets

The experimental procedure underwent review and approval by the Animal Care and Use Committee of Tianjin Agricultural University, Tianjin, China (approval number: 2023LLSC25). This study utilized a sample of 2,700 female quails at 28 weeks of age, with an average weight of 174.42±1.44 g, which were randomly assigned to three groups of 900 quails each. Each group was further divided into nine replicates of 100 quails. The groups were designated as group C (control), T1, and T2. The control group received a basal diet, whereas the T1 and T2 groups received a basal diet supplemented with 0.05% and 0.1% ABP, respectively. The basal diet was formulated based on the recommendations of the NRC (1994), as presented in Table 1. All quails were housed in a hygienic and temperature-controlled (29°C±1°C) environment. They were exposed for 16 h lighting program per day throughout the experimental period and had ad libitum access to feed and water.

### Productive performance and egg parameters

The feed conversion ratio was calculated as the ratio of feed intake (in grams) to egg weight (in grams). Daily monitoring was conducted for feed intake, laying rate, number of eggs, egg weight, and mortality rate.


Laying rate=(number of eggs laid/number of birds)×100%

At the end of the 31st and 34th week of the experiment, 45 eggs from each treatment replicate were randomly collected to determine egg and eggshell quality. After weighing and measuring length and width, the eggs were carefully broken on a glass plate to measure internal and external quality. Yolks were separated from albumen and weighed. The weight of the albumen was obtained by subtracting the weight of the egg yolk and shell from the weight of the egg. Yolk and albumen weights were expressed as a percentage of the whole egg. Shell thickness (without membrane) was measured at three locations (air cell, equator, and sharp end) using a micrometer and averaged. Yolk diameter and height were measured with a vernier caliper. The yolk index was calculated by dividing yolk height by yolk diameter, while the egg shape index was calculated as the ratio of egg width to length. The Hough unit (HU) score was calculated using the following equation:


$$\text{HU }(\%)=100\times \text{log }(\text{H}+7.57-1.7\times {\text{W}}^{0.37})$$

where H and W refer to albumen height and egg weight, respectively. Yolk color was determined using a yolk color fan with a 1 to 15 scale. The inferior egg rate was obtained by observing the ratio of inferior egg production to the total number of eggs produced each day.

### Carcass parameters

At the end of the experiment, nine quails from each treatment group were randomly selected and sacrificed to determine carcass parameters. The quails were fasted for 12 hours before being slaughtered. After bleeding, the weights of the carcass, heart, liver, spleen, lung, kidney, gizzard, proventriculus, breast, and thigh were recorded, and the corresponding percentages (% of weight) were calculated.

### Intramuscular fatty acid profile

The fatty acid profiles were analyzed using breast muscle samples. Fatty acids were extracted and methylated in a single tube using the direct methylation method with some modifications [9]. In brief, 0.5 mg of tridecanoic acid was added to the samples in a 15 mL glass tube, followed by the addition of 5.3 mL of methanol and 700 μL of 10 N KOH. The tube was then incubated in a 55°C water bath for 1.5 hours with brief vortexing every 20 minutes. Next, the tube was cooled to room temperature and 580 μL of 24 N H_2_SO_4_ was added. The incubation and cooling steps were repeated. Finally, 3 mL of hexane was added, and the sample was vortexed for five minutes and centrifuged at 3,000 rpm for five minutes. The upper phase was transferred to a GC vial (Agilent, Santa Clara, CA, USA) and analyzed using gas chromatography-flame ionization detection (GC-FID; Agilent 7890B, Santa Clara, CA, USA) and SP-2560 (100 m × 0.25 mm, length × internal diameter; 0.2 μm, df; Sigma-Aldrich, St. Louis, MO, USA). FAME 37 was used as a reference for peak identification. The operating conditions for GC-FID followed the FAME37 manual, with a column oven temperature of 140°C for five minutes, a ramp of 4°C/min to 240°C, and holding at 240°C for 28 minutes. The injector and detector temperatures were maintained at 260°C, and the split ratio was 1:30. The injection volume was 1 μL.

### Serum metabolites, immune response parameters and antioxidative properties

At the end of the experiment, blood samples were collected from nine quails randomly selected from each replicate. Blood was collected from the branchial vein and then centrifuged at 2,400 g for seven minutes at 4°C. The serum was collected and stored at −20°C until use. Commercial kits from Biosino Bio-Technology and Science Incorporation in Beijing, China were used to determine the serum contents of several parameters, including glucose (GLU), albumin (ALB), alkaline phosphatase (ALP), aspartate aminotransferase (AST), cholesterol (CHO), high-density lipoprotein (HDL), low-density lipoprotein (LDL), triglyceride (TG), total protein (TP), uric acid (UA), and urea. Commercial kits from InTec Products, Inc. in Xiamen, China were used to determine serum immunoglobulin A (IgA) and immunoglobulin M (IgM). Additionally, commercial kits from Nanjing Jiancheng in Nanjing, China were used to determine the antioxidative capacity index glutathione peroxidase (GSH-Px), total antioxidant capacity colorimetric (T-AOC), and total superoxide dismutase (T-SOD).

### Intestinal morphology

At the end of the experiment, segments of the duodenum, jejunum, and ileum (2 cm) from nine quails per treatment group were excised for morphology analysis. The segments were carefully washed with phosphate-buffered saline to avoid damage to the intestinal tissue and then collected and fixed in 10% neutral buffered formalin solution for 24 hours. The segments were then embedded in paraffin and sectioned. Hematoxylin-eosin (H&E) staining was performed on the sections according to the methods described by Bancroft and Gamble [10]. Images of the sections were captured using an ECHO Revolve microscope at 10× magnification, and villi height and crypt depth (CD) were measured using Revolve-Pro software.

### Gut microbiome

At the end of the experiment, fresh cecal contents were collected from each treatment group. The bacterial genomic DNA was extracted from the frozen fecal samples, which were previously stored at −80°C. To investigate the gut microbial community of quails, the V3+V4 region of the bacterial 16S rRNA gene was amplified by polymerase chain reaction (PCR) using specific primers (forward primer: 5’-ACTCCT ACGGGAGGCAGCA-3’; reverse primer: 5’-GGACTACH VGGGTWTCTAAT-3’). The raw paired-end reads obtained from the original DNA were merged using FLASH32 and assigned to each sample based on a unique barcode.

High-throughput pyrosequencing of the PCR products was conducted on an Illumina MiSeq platform at Biomarker Technologies Co. Ltd. in China.

The resulting high-quality sequences were analyzed using the Quantitative Insights Into Microbial Ecology (QIIME, v1.8.0) software. The sequences were clustered into operational taxonomic units (OTUs) using UCLUST at 97% similarity. Subsequently, the OTUs were taxonomically classified using the RDP Classifier against a curated Green Genes database with a bootstrap cutoff of 80%.

### Statistical analysis

One-way analysis of variance was performed using SPSS for Windows version 23.0 to statistically analyze the data. Duncan’s multiple-range test was employed to identify significant differences among the treatments. The level of statistical significance was set at p<0.05.

## RESULTS

### Productive performance

Table 2 suggests that adding ABP to the Korean quail diet did not significantly affect their average daily feed intake. However, the T1 group, which received a diet supplemented with 0.05% (w/w) ABP, had a higher average daily egg yield and improved F/E (feed intake [g] / egg weight [g]) compared to the other treatment groups (p<0.01). Notably, the T1 group had the highest laying rate among all the treatment groups. It is worth mentioning that the T2 group had a significantly higher mortality rate compared to the control group (p<0.01), while the T1 group had the lowest mortality rate.

### Egg quality and carcass characteristics

A total of 270 quail eggs were collected, and their quality characteristics were measured. At the third and sixth week of the trial, there were no significant differences in most egg quality characteristics (Table 3) between the treatment groups, including egg shape index, yolk index, yolk color, yolk rate, and albumen rate. However, at the third week of the trial, the Haugh unit of T1 group eggs was significantly higher than that of C and T2 groups (p<0.01), and the rate of inferior eggs in the experimental group with ABP added to the diet was significantly lower than that in the control group (p<0.01). At the sixth week of the trial, the average egg weight of group T1 was significantly lower than that of the control group (p<0.05), and the eggshell thickness of group T2 was significantly lower than that of the other groups (p<0.01). The results for inferior egg rate were the same as in the third week.

The results for carcass characteristics and the relative weight of internal organs are presented in Supplementary Table S1. No significant effects were observed among the different treatments in these carcass traits.

### Serum metabolites, immune response parameters and antioxidative properties

Table 4 summarizes the blood biochemical indicators, immune response parameters, and some antioxidant capacity indicators. The results suggest that quails fed with 0.05% (w/w) ABP had a significantly higher concentration of total protein compared to the other groups (p<0.05). Additionally, serum LDL-C levels were significantly decreased in quails that received ABP compared to the control group (p<0.05).

No significant effects of the dietary supplements on IgA and IgM levels, which are indicators of immune response, were found. However, the study did find that dietary supplementation with ABP improved antioxidant properties, as indicated by an increase in GSH-Px, T-SOD (p<0.05), and T-AOC levels (p<0.05).

### Intestinal morphology

Supplementary Figure S1 and Table 5 show that microscopic examination of the small intestine revealed no histopathological lesions in all treatment groups. Dietary supplementation did not affect the morphology of the duodenum. However, in the jejunum, quails fed with ABP showed a significant increase in villus height (VH) compared to the control group (p<0.05), which resulted in an increased villus to crypt ratio (p<0.05).

### Intramuscular fatty acid profile

The addition of ABP significantly altered the composition of intramuscular fatty acids, as shown in Table 6. Compared to the control group, the addition of ABP significantly increased the concentration of C14:0, C15:0, C16:0, and C18:0, with the exception of C18:0 being dose-dependent (p<0.01). The addition of ABP did not change the concentration of C13:0 and C20:0. For monounsaturated fatty acids (MUFA) (C14:1, C16:1, C18:1, C20:1), the addition of ABP also dose-dependently increased the concentration in intramuscular fat (p<0.01). PUFA levels were also affected by the diet; the concentration of C18:2 and C18:3 in the T2 group was significantly higher than in T1 and C groups (p<0.01). In addition, the T1 group had higher concentrations of C20:2, C20:4, and C22:6 compared to the T2 group (p<0.01). In general, the addition of ABP significantly increased the content of saturated fatty acids (SFA), MUFA, and PUFA in intramuscular fat, and the experimental group added with 0.05% ABP had the highest PUFA content.

### Gut microbiome

**Figure 1:**
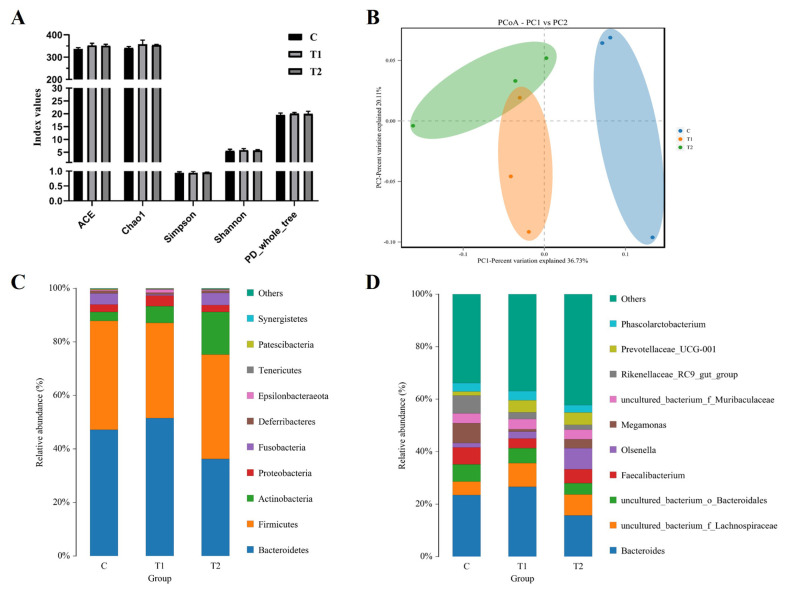
Effect of Agaricus blazei polysaccharide on gut microbiota of Korean quail. Alpha diversity indices per group (A), principal coordinate analysis (PCoA) plot (B), overall compositions of the gut microbiota at the phylum level (C), and genus level (D). C, quails fed basal diets; T1, quails fed basal diet with 0.05% (w/w) Agaricus blazei polysaccharide; T2, quails fed basal diet with 0.1% (w/w) Agaricus blazei polysaccharide.

The impact of ABP on gut microbiota was evaluated through 16S rRNA sequencing analysis to explore the microbial community dynamics. Alpha diversity indices, including ACE, Chao1, Simpson, Shannon, and PD, demonstrated similar patterns in all three groups, suggesting that the addition of ABP did not alter alpha diversity (Figure 1A). To further investigate the changes in gut microbiota, principal coordinate analysis (PCoA) was conducted based on weighted UniFrac distances, revealing that ABP significantly affected the gut microbiota. The results showed that samples were clustered into two distinct groups (C vs T1 and T2), and the samples of T1 and T2 were also distinguished from each other (Figure 1B).

Furthermore, the relative abundance of microbial taxa in each group was compared, and several phyla and genera were found to be significantly different. At the phylum level, all groups shared the following ten phyla: Bacteroidetes, Firmicutes, Actinobacteria, Proteobacteria, Fusobacteria, Deferribacteres, Epsilonbacteraeota, Tenericutes, Patescibacteria and Synergistetes (Figure 1C). The three dominant phyla, containing more than 85% of the total 16S rRNA gene sequences, were Bacteroidetes, Firmicutes, and Actinobacteria. In particular, Actinobacteria was more abundant in the T2 group than in the C or T1 group (Supplementary Table S2).

At the genus level, three dominant genera containing more than 30% of the total 16S rRNA gene sequences were i) C group: Bacteroides, Megamonas and Rikenellaceae_RC9_gut_group; ii) T1 group: Bacteroides, uncultured_bacterium_f_Lachnospiraceae and uncultured_bacterium_o_Bacteroidales; iii) T3 group: Bacteroides, Olsenella and uncultured_bacterium_f_Lachnospiraceae (Figure 1D; Supplementary Table S3). In particular, Prevotellaceae_UCG-001 was more abundant in T1 and T2 than C.

## DISCUSSION

Nutritional and functional feed additives play a crucial role in enhancing the productivity of livestock animals. This study aimed to examine the impact of ABPs on production performance, egg quality, blood biochemistry, intestinal morphology, and intestinal flora in Korean quail.

The supplementation of 0.05% ABP has shown significant improvements in average daily egg production, egg production rate, feed-egg ratio, and a noticeable decrease in the incidence of dead scouring. Currently, there is a lack of research investigating the potential of ABP additives to enhance the production efficiency of broilers or laying hens. Furthermore, no studies have been conducted on the effects of ABP additives in quail. However, multiple studies conducted on chickens have provided confirmation that ABP can alleviate oxidative stress induced by cadmium. This effect is achieved by improving antioxidant capacity and reducing inflammation. Additionally, the incorporation of Agaricus blazei into the broiler diet demonstrated immunostimulatory activity and a hypocholesterolemic effect [11,12]. The relatively high production performance observed in the treatment group supplemented with ABPs can be attributed to its diverse biological activities, which enhance the immunity of quails and help maintain their overall health [13]. The HU serves as a crucial indicator for assessing the shelf life and freshness of poultry eggs. It is directly correlated with egg weight and protein quality, making it an essential parameter. Apart from disease, the age of laying birds emerges as the primary factor influencing the protein quality of freshly laid eggs. With advancing age, the initial protein content experiences a rapid decline, leading to a decrease in HU and an escalation in the score’s variability [14]. In this experiment, the supplementation of 0.05% ABP led to a significant increase in the HU of quail eggs during the third week. This observation suggests that ABP may mitigate the decline in protein quality associated with the aging of quail. However, the inclusion of 0.1% ABP did not yield a similar effect. The specific reasons behind this discrepancy require further investigation in subsequent experiments.

It is widely acknowledged that during the late laying period, the ovary and other functions of poultry gradually decline. Consequently, the hatching ability is significantly reduced, and both the internal and external quality traits of poultry eggs deteriorate considerably with the age of the flock [15]. Moreover, the prevailing breeding mode for laying quails is primarily characterized by intensive and high-density farming practices. Quails are highly susceptible to external factors and consistently experience stress, consequently impacting the quality of their eggs. As a result, the rate of unqualified eggs tends to rise. However, in this experiment, the addition of ABP during the third week substantially reduced the rate of unqualified quail eggs. Furthermore, even during the sixth week, the rate of unqualified eggs remained lower than that of the control group.

Various factors have been reported to significantly influence the serum biochemical parameters in livestock and poultry. These factors include feed additives, genotype, and ambient temperature. GLU can provide a portion of the body’s energy requirements, while TP serves various functions in the body, including maintaining osmotic pressure in poultry and facilitating nutrient transport [16]. It is widely accepted that both GLU and TP are limiting factors that significantly impact livestock production. In the experiments conducted in this study, the TP levels in the T1 group were notably higher compared to the control group, consistent with the previous production performance results [17]. The inclusion of 0.05% ABP enhanced the synthesis and deposition of proteins in the body. Li et al [18] conducted a study utilizing SD rats to establish a hyperlipidemia model through a long-term high-fat diet. The researchers observed a notable increase in the liver and spleen indexes among the rats in the model group. However, following an 8-week gavage treatment of ABP, the serum levels of TC, TG, and LDL-C exhibited significant reductions compared to the model group. Conversely, the level of HDL-C displayed a significant increase. Moreover, there was a significant decrease in the liver and spleen indexes. These findings closely corresponded to the outcomes observed in the ABP-added group of the present experiment.

The concentration of polysaccharides is strongly correlated with the capacity to scavenge free radicals and exhibit reducing ability. Higher concentrations of polysaccharides correspond to greater antioxidant capacity [19]. In the current experiment, the supplementation of ABP led to an augmentation in the activity of quail’s endogenous antioxidant enzymes, which exhibited a dose-dependent increase.

Intestinal morphology plays a crucial role in nutrient absorption and growth performance among animals. Notable alterations in intestinal morphology, such as villi atrophy and crypt hyperplasia, can directly result in malabsorption, diarrhea, and growth inhibition in livestock [20]. Moreover, the villus VH/CD ratio serves as a vital indicator for assessing the impact of small intestine morphology, reflecting the digestive and absorptive capacity of nutrients [21]. The supplementation of 0.05% ABP resulted in a significant increase in VH within the jejunum and an elevation in the VH/CD ratio, indicating that ABP has the potential to stimulate the proliferation, differentiation, and migration of intestinal epithelial cells. Consequently, ABP contributes to the enhancement of intestinal morphology and mucosal barrier function.

Quail meat contains higher levels of protein and essential fatty acids compared to chicken [22], while exhibiting lower levels of SFAs [23]. Additionally, quail has a shorter generation interval, smaller body size, and requires less space and food compared to chickens [24], making it an accessible source of high-quality animal protein. In this experiment, the addition of ABP to the quail’s diet significantly increased the content of SFAs, MUFAs, and PUFAs in the intramuscular fat. Notably, there was a significant increase in the content of n-3 PUFA, which plays a crucial role in various physiological functions. While these findings provide a theoretical basis for the production of functional livestock products, further exploration is necessary to assess the impact on meat quality.

The gut microbiota plays a crucial role in the growth and health of the host by performing essential functions such as metabolism, immune response, and protection [25]. In the past, antibiotics were commonly used in livestock and poultry farming to manage sudden animal diseases. However, studies have demonstrated that antibiotic treatment can significantly impact the gut microbiota. Antibiotics can disrupt the balance of the gut, leading to alterations of up to 90% in metabolites such as bile acids, eicosanoids, and steroid hormones. These disruptions in metabolic pathways can have profound effects on the host [26]. Numerous studies have shown that polysaccharides have the potential to improve the intestinal microbiota by increasing species abundance and maintaining dynamic balance among the flora [27–29]. However, the addition of ABP did not induce a significant change in alpha diversity, which could be attributed to its inherent antibacterial or antifungal properties [30,31]. The results of Beta diversity analysis revealed significant differences between the ABP treatment group and the control group, suggesting that ABP has the potential to alter the species composition of the quail intestinal flora.

At the genus level, ABP treatment had an impact on multiple genera. Notably, the ABP-added group exhibited increased abundance of uncultured_bacterium_f_Lachnospiraceae and Prevotellaceae_UCG-001 compared to the control group. The uncultured_bacterium_f_Lachnospiraceae is a group of spore-forming bacteria that ferment a wide range of plant polysaccharides into short-chain fatty acids, including butyrate and acetate. Butyrate has been reported as a significant nutrient source for colonic epithelial cells. Prevotellaceae_UCG-001 belongs to the anaerobic Gram-negative bacteria of the Bacteroidetes phylum, which also comprises the clinically significant genera Bacteroides and Porphyromonas [32,33]. Currently, the prevailing explanation suggests that greater diversity of Prevotella correlates with enhanced fermentation ability of the microbiota, leading to increased benefits for animal intestinal health [34,35]. In contrast, the ABP-added group exhibited a decrease in Megamonas and Rikenellaceae_RC9_gut_group. According to a previous study, Megamonas functions as a hydrogen sink in the ceca of broilers, leading to increased production of short-chain fatty acids, specifically acetate [36]. Furthermore, additional studies have demonstrated that the genus Megamonas contributes to the P461-PWY metabolic pathway, which results in the production of acetate. This process, in turn, promotes TG accumulation and ultimately contributes to the development of non-alcoholic fatty liver disease [37,38]. The results of carcass characteristics revealed that the addition of ABP led to a decrease in the liver index, possibly associated with a reduction in liver TG accumulation. The previous study revealed a significant increase in the Rikenellaceae_RC9_gut_group genus in the high-fat diet group with high-dose genistein in mice and rats with an ISO-induced acute myocardial ischemia model [39,40]. This genus may have an important role in lipid metabolism. The inclusion of ABP in this study resulted in a reduction of lipid metabolism-related markers, including serum TG and LDL, and showed a positive correlation with the Rikenellaceae_RC9_gut_group. Due to limitations, a total of three fecal samples were collected from each experimental group for the analysis of the intestinal flora. However, the limited number of samples resulted in the absence of significant changes in the data. Importantly, acquiring a larger number of samples will be essential to comprehensively evaluate and confirm the impact of ABP on the intestinal flora.

The results of the present study suggest that dietary supplementation of ABP improved egg production rate, egg quality, antioxidant function, and jejunal VH/CD ratio. Furthermore, it resulted in changes in intramuscular fat, specifically an increase in PUFA content. These indicators support the potential of ABP as a viable feed additive in quail diets, with the 0.05% concentration showing greater effectiveness.

## Figures and Tables

**Table 1:** Nutrient levels in the experimental diets, %, as fed basis

Items	Group^1)^		

	C	T1	T2
Corn	59.00	59.00	59.00
Soybean meal	21.30	21.30	21.30
Imported fish meal	5.00	5.00	5.00
Limestone	5.00	5.00	5.00
Wheat Bran	3.00	3.00	3.00
Vegetable oil	3.00	3.00	3.00
CaHPO_4_	1.00	1.00	1.00
Salt	0.35	0.35	0.35
Lysine	0.05	0.05	0.05
DL-methionine	0.30	0.30	0.30
Premix^2)^	2.00	2.00	2.00
ABP	0	0.05	0.1
Nutrient levels^3)^ of basal diet			
Metabolic energy (MJ/kg)		2.82	
Crude protein		20.00	
Calcium		2.38	
Available phosphorus		0.42	
Lysine		1.00	
Methionine		0.45	

1) C, quails fed basal diets; T1, quails fed basal diet with 0.05% (w/w) Agaricus blazei polysaccharide; T2, quails fed basal diet with 0.1% (w/w) Agaricus blazei polysaccharide.

2) The premix provided the following per kg of diets: vitamins A 8,000 IU, vitamins B_1_ (thiamine) 1 mg, vitamins B_2_ (riboflavin) 4 mg, vitamins B_6_ (pyridoxine) 1.5 mg, vitamins B_12_ (cobalamin) 0.12 mg, vitamins D_3_ (cholecalciferol) 1,500 IU, vitamins E 30 IU, vitamins K_3_ (menadione) 2 mg, Biotin 0.5 mg, D-pantothenic acid 16.5 mg, Nicotinic acid 20 mg, Cu (as copper sulfate) 5 mg, Fe (as ferrous sulfate) 40 mg, Mn (as manganese sulfate) 50 mg, Zn (as zinc sulfate) 30 mg, I (as potassium iodide) 1 mg, Se (as sodium selenite) 0.40 mg.

3) ME, lysine and methionine were a calculated value, while crude protein, calcium and total phosphorus was calculated values.

**Table 2:** Effect of Agaricus blazei polysaccharide on productive performance of Korean quail

Items	Groups^1)^		

	C	T1	T2
Average daily feed intake (g/d)	22.45±0.21	22.28±0.13	22.23±0.07
Average daily egg yield (g/d)	8.51±0.03^b^	8.80±0.03^a^	8.51±0.03^b^
F/E	2.62±0.03^a^	2.52±0.01^b^	2.58±0.02^a^
Laying rate (%)	76.97±0.28^b^	79.42±0.26^a^	77.32±0.25^b^
Mortality %	0.26±0.03^b^	0.19±0.03^b^	0.39±0.04^a^

Values are means±standard error of the mean (n = 9).

F/E, feed intake (g) to egg weight (g) ratio.

1) C, quails fed basal diets; T1, quails fed basal diet with 0.05% (w/w) Agaricus blazei polysaccharide; T2, quails fed basal diet with 0.1% (w/w) Agaricus blazei polysaccharide.

a,b Means within the same row with no common superscripts differ significantly (p<0.05).

**Table 3:** Effect of Agaricus blazei polysaccharide on egg quality of Korean quail

Items	Groups^1)^		

	C	T1	T2
The 3rd week			
Average egg weight (g)	11.63±0.13	11.51±0.15	11.45±0.16
Egg shape index (%)	1.29±0.01	1.29±0.01	1.28±0.01
Eggshell thickness (mm)	0.22±0.00	0.21±0.00	0.21±0.00
Yolk index (%)	0.51±0.01	0.50±0.01	0.51±0.01
Yolk color	3.86±0.15	3.67±0.14	3.92±0.17
Haugh unit	54.52±0.29^b^	58.14±0.26^a^	54.35±0.31^b^
Yolk rate (%)	30.23±0.32	29.59±0.74	29.99±0.40
Albumen rate (%)	59.45±0.41	59.65±0.89	59.35±0.47
Eggshell rate (%)	10.32±0.25	10.77±0.30	10.66±0.30
Unqualified egg rate (%)	1.18±0.10^a^	0.53±0.05^b^	0.66±0.07^b^
The 6th week			
Average egg weight (g)	12.73±0.24^a^	11.96±0.13^b^	12.52±0.23^ab^
Egg shape index (%)	1.28±0.01	1.28±0.01	1.29±0.02
Eggshell thickness (mm)	0.30±0.01^a^	0.28±0.01^a^	0.25±0.01^b^
Yolk index (%)	0.47±0.01	0.46±0.01	0.44±0.01
Yolk color	6.29±0.30	6.14±0.23	5.47±0.29
Haugh unit	52.99±0.39	53.99±0.31	53.23±0.43
Yolk rate (%)	32.15±1.26	30.84±1.18	28.48±1.27
Albumen rate (%)	56.44±2.09	57.03±0.49	59.71±1.62
Eggshell rate (%)	10.49±0.72	10.34±0.26	10.13±0.38
Unqualified egg rate (%)	1.01±0.06^a^	0.60±0.05^b^	0.65±0.07^b^

Values are means±standard error of the mean (n = 45).

1) C, quails fed basal diets; T1, quails fed basal diet with 0.05% (w/w) Agaricus blazei polysaccharide; T2, quails fed basal diet with 0.1% (w/w) Agaricus blazei polysaccharide.

a,b Means within the same row with no common superscripts differ significantly (p<0.05).

**Table 4:** Effects of Agaricus blazei polysaccharide on serum metabolites, immune response parameters and antioxidative properties of Korean quail

Items	Groups^1)^		

	C	T1	T2
Serum metabolites			
ALP (U/L)	367.78±50.22	392.18±24.81	341.64±37.24
AST (U/L)	345.84±18.83	397.28±41.37	362.40±22.92
GLU (mmol/L)	13.76±0.80	12.79±0.67	12.23±0.44
TP (g/L)	36.67±2.64^b^	47.36±2.73^a^	35.65±3.83^b^
Alb (U/L)	15.38±1.23	17.02±1.23	13.62±1.27
UA (umol/L)	66.85±9.06	65.78±10.89	61.67±9.74
UREA (mg/dL)	1.21±0.12	1.45±0.24	1.30±0.21
CHO (mmol/L)	4.58±0.70	3.97±1.02	3.80±0.49
TG (mmol/L)	6.85±0.90	5.88±0.46	7.55±1.24
HDL-C (mmol/L)	1.66±0.22	2.18±0.19	1.71±0.16
LDL-C (mmol/L)	1.85±0.52^a^	0.51±0.07^b^	0.65±0.23^b^
Immune response parameters			
IgA (g/L)	0.22±0.02	0.22±0.02	0.25±0.01
IgM (g/L)	0.37±0.04	0.42±0.02	0.45±0.02
Antioxidative properties			
GSH-Px (U/mL)	855.37±101.65	1,169.59±74.63	1,056.20±35.38
T-SOD (U/mL)	155.12±0.85^b^	155.30±1.03^b^	166.72±0.23^a^
T-AOC (mmol/L)	0.57±0.02^b^	0.62±0.03^b^	0.68±0.02^a^

Values are means±standard error of the mean (n = 9).

ALP, alkaline phosphatase; AST, aspartate transaminase; GLU, glucose; TP, total protein; Alb, albumin; UA, uric acid; TCHO, total cholesterol; TG, triglyceride; HDL-C, high-density lipoprotein-cholesterol; LDL-C, low density lipoprotein cholesterol; IgA, immune globulin A; IgM, immune globulin M; GSH-Px, glutathione peroxidase; T-SOD, total superoxide dismutase; T-AOC, total antioxidant capacity colorimetric.

1) C, quails fed basal diets; T1, quails fed basal diet with 0.05% (w/w) Agaricus blazei polysaccharide; T2, quails fed basal diet with 0.1% (w/w) Agaricus blazei polysaccharide.

a,b Means within the same row with no common superscripts differ significantly (p<0.05).

**Table 5:** Effect of Agaricus blazei polysaccharide on intestinal morphology of Korean quail

Items	Groups^1)^		

	C	T1	T2
Total intestinal length (cm)	66.80±2.15	66.82±1.56	66.06±1.92
Villus height (VH, μm)			
Duodenum	702.40±29.18	721.28±28.55	702.91±34.96
Jejunum	589.07±7.37^b^	726.66±21.08^a^	687.28±26.72^ab^
Ileum	453.24±12.43	436.94±8.08	449.52±11.97
Crypt depth (CD, μm)			
Duodenum	120.04±10.06^ab^	94.53±3.73^b^	117.69±4.17^a^
Jejunum	120.17±9.25	112.37±6.24	98.08±4.95
Ileum	83.44±3.35	76.40±1.48	79.65±4.21
VH/CD			
Duodenum	6.08±0.60	7.64±0.23	6.42±0.37
Jejunum	4.95±0.30^b^	6.36±0.33^a^	7.05±0.32^a^
Ileum	5.71±0.28	5.68±0.13	5.57±0.52

Values are means±standard error of the mean (n = 9).

1) C, quails fed basal diets; T1, quails fed basal diet with 0.05% (w/w) Agaricus blazei polysaccharide; T2, quails fed basal diet with 0.1% (w/w) Agaricus blazei polysaccharide.

a,b Means within the same row with no common superscripts differ significantly (p<0.05).

**Table 6:** Effect of Agaricus blazei polysaccharide on intramuscular fatty acids in Korean quail

Items	Groups^1)^		

	C	T1	T2
Tridecanoic acid C13:0 (%)	0.098±0.004	0.085±0.001	0.097±0.001
Myristic acid C14:0 (%)	0.035±0.001^c^	0.045±0.001^b^	0.047±0.001^a^
Pentadecanoic acid C15:0 (%)	0.009±0.000^b^	0.011±0.000^a^	0.011±0.000^a^
Palmitic acid C16:0 (%)	2.025±0.003^c^	2.769±0.003^b^	2.900±0.002^a^
Stearic acid C18:0 (%)	0.309±0.001^c^	0.426±0.012^a^	0.392±0.002^b^
Arachidic acid C20:0 (%)	0.006±0.000	0.007±0.000	0.011±0.003
Myristoleic acid C14:1 (%)	0.004±0.000^c^	0.006±0.000^b^	0.008±0.000^a^
Palmitoleic acid C16:1 (%)	0.148±0.001^c^	0.202±0.001^b^	0.224±0.003^a^
Oleic acid C18:1 (%)	1.969±0.003^c^	2.719±0.002^b^	2.750±0.003^a^
Gondoic acid C20:1 (%)	0.017±0.001^b^	0.025±0.002^b^	0.035±0.004^a^
Linoleic acidC18:2 (%)	1.931±0.003^c^	2.474±0.003^b^	2.525±0.001^a^
α-Linolenic acid C18:3n-3 (%)	0.068±0.000^c^	0.084±0.000^b^	0.097±0.000^a^
Eicosadienoic acid C20:2 (%)	0.022±0.002^a^	0.020±0.002^a^	0.014±0.001^b^
Dihomogamma-linolenic acid C20:3n-6 (%)	0.009±0.000	0.009±0.000	0.009±0.000
Arachidonic acid C20:4n-6 (%)	0.224±0.006^c^	0.329±0.008^a^	0.257±0.002^b^
Docosahexaenoic acid			
C22:6n-3 (%)	0.072±0.000^c^	0.111±0.000^a^	0.104±0.001^b^
SFA (%)	2.479±0.004^c^	3.371±0.004^b^	3.452±0.006^a^
MUFA (%)	2.139±0.004^c^	2.952±0.005^a^	3.017±0.010^a^
PUFA (%)	2.340±0.003^c^	3.050±0.003^a^	3.003±0.002^b^

Values are means±standard error of the mean (n = 9).

SFA, saturated fatty acids; MUFA, monounsaturated fatty acids; PUFA, polyunsaturated fatty acids.

1) C, quails fed basal diets; T1, quails fed basal diet with 0.05% (w/w) Agaricus blazei polysaccharide; T2, quails fed basal diet with 0.1% (w/w) Agaricus blazei polysaccharide.

a–c Means within the same row with no common superscripts differ significantly (p<0.05).

## SUPPLEMENTARY MATERIAL

**Supplementary Table S1**. Effect of Agaricus blazei polysaccharide on carcass characteristics of Korean quail.

**Supplementary Table S2**. Species annotation and taxonomic analysis of cecal contents of Korean quail (phylum level)

**Supplementary Table S3**. Species annotation and taxonomic analysis of cecal contents of Korean quail (genus level)

**Supplementary Figure S1**. Effect of Agaricus blazei polysaccharide on intestinal morphology of Korean quail (10×, scale bar 200 μm).
