# Supplementary material for: Effects of dietary supplementation of polysaccharide from Agaricus blazei Murr on productive performance, egg quality, blood metabolites, intestinal morphology and microbiota of Korean quail
Source: Anim Biosci. 2024 Apr 1;37(8):1452–62. doi: 10.5713/ab.23.0441 (PMC11222865; doi:10.5713/ab.23.0441)
Supplement: Supplementary file 1 [file ab-23-0441-Supplementary-Table-1.pdf]

Effects of dietary supplementation of polysaccharide from Agaricus blazei Murr on productive performance, egg quality, blood metabolites, intestinal morphology and microbiota of Korean quail

Liang Hong, Zheng Ma et al.

Supplementary Information

Table S1. Effect of Agaricus blazei polysaccharide on carcass characteristics of Korean quail.

Items	Groups			*p*-value
	C	T1	T2	
Dressed carcass %	94.12±0.82	93.97±1.13	95.14±0.45	0.566
Half evisceration yield %	78.62±1.51	78.23±1.05	77.16±1.41	0.730
Eviscerated yield %	62.21±1.46	63.75±1.12	60.97±1.18	0.305
Breast muscle %	26.44±0.88	25.15±0.81	26.99±1.47	0.467
Leg muscle %	15.06±0.59	13.87±0.63	14.24±0.45	0.327
Abdominal fat %	4.56±0.81	4.89±0.29	4.91±0.96	0.930
Heart %	0.78±0.05	0.76±0.07	0.82±0.04	0.706
Liver %	2.57±0.18	2.38±0.11	2.37±0.10	0.527
Spleen %	0.10±0.03	0.06±0.01	0.08±0.01	0.479
Lung %	0.80±0.03	0.71±0.04	0.67±0.06	0.194
Kidney %	0.75±0.09	0.56±0.06	0.60±0.06	0.151
Muscular stomach %	1.66±0.04	1.73±0.18	1.59±0.06	0.565
Glandular stomach %	0.41±0.02	0.38±0.02	0.41±0.02	0.414

C: quails fed basal diets, T1: quails fed basal diet with 0.05% (w/w) Agaricus blazei polysaccharide, T2: quails fed basal diet with 0.1% (w/w) Agaricus blazei polysaccharide, Values are means ± SEM (n=9).
