# Supplementary material for: Effects of dietary supplementation of polysaccharide from Agaricus blazei Murr on productive performance, egg quality, blood metabolites, intestinal morphology and microbiota of Korean quail
Source: Anim Biosci. 2024 Apr 1;37(8):1452–62. doi: 10.5713/ab.23.0441 (PMC11222865; doi:10.5713/ab.23.0441)
Supplement: Supplementary file 2 [file ab-23-0441-Supplementary-Table-2.pdf]

Table S2. Species annotation and taxonomic analysis of cecal contents of Korean quail (phylum level).

Items	Groups			*p*-value
	C	T1	T2	
Bacteroidetes	46.62±4.35	53.29±5.96	36.16±4.17	0.118
Firmicutes	40.63±2.83	33.71±6.23	39.19±2.57	0.513
Actinobacteria	3.42±1.42	5.90±2.55	16.23±6.99	0.170
Proteobacteria	2.78±0.46	4.19±1.27	2.54±0.46	0.372
Fusobacteria	4.60±3.99	0.42±0.17	4.17±3.92	0.533
Deferribacteres	0.98±0.72	0.79±0.40	0.82±0.43	0.967
Epsilonbacteraeota	0.36±0.24	1.23±0.75	0.29±0.08	0.577
Tenericutes	0.15±0.03	0.28±0.10	0.27±0.12	0.580
Patescibacteria	0.24±0.21	0.11±0.03	0.11±0.08	0.857
Synergistetes	0.19±0.07	0.01±0.01	0.13±0.12	0.212
Others	0.03±0.01	0.07±0.03	0.10±0.02	0.124

C: quails fed basal diets, T1: quails fed basal diet with 0.05% (w/w) Agaricus blazei polysaccharide, T2: quails fed basal diet with 0.1% (w/w) Agaricus blazei polysaccharide, Values are means ± SEM (n=3).
