# Supplementary material for: Effects of dietary supplementation of polysaccharide from Agaricus blazei Murr on productive performance, egg quality, blood metabolites, intestinal morphology and microbiota of Korean quail
Source: Anim Biosci. 2024 Apr 1;37(8):1452–62. doi: 10.5713/ab.23.0441 (PMC11222865; doi:10.5713/ab.23.0441)
Supplement: Supplementary file 3 [file ab-23-0441-Supplementary-Table-3.pdf]

Table S3. Species annotation and taxonomic analysis of cecal contents of Korean quail (genus level).

Items	Groups			*p*-value
	C	T1	T2	
Bacteroides	22.51±8.51	28.16±7.30	15.72±0.66	0.447
uncultured_bacterium_f_ Lachnospiraceae	5.34±2.02	8.19±2.89	7.94±0.81	0.593
uncultured_bacterium_o_ Bacteroidales	6.54±3.02	5.86±0.55	4.21±0.85	0.407
Faecalibacterium	6.31±2.11	3.45±1.28	5.40±3.93	0.752
Olsenella	1.72±0.78	2.58±1.25	8.37±4.79	0.494
uncultured_bacterium_f_ Muribaculaceae	3.75±0.19	3.76±0.94	3.78±0.95	0.946
Megamonas	7.15±3.62	0.74±0.54	3.22±1.85	0.234
Rikenellaceae_RC9_gut_group	7.23±3.74	2.58±1.46	1.80±1.20	0.301
Prevotellaceae_UCG-001	1.52±0.78	4.35±0.68	4.63±1.72	0.191
Phascolarctobacterium	3.20±1.36	3.28±1.70	2.86±1.62	0.980
Others	34.71±6.84	37.04±2.75	42.07±6.18	0.652
