# Supplementary material for: Effects of dietary supplementation of polysaccharide from Agaricus blazei Murr on productive performance, egg quality, blood metabolites, intestinal morphology and microbiota of Korean quail
Source: Anim Biosci. 2024 Apr 1;37(8):1452–62. doi: 10.5713/ab.23.0441 (PMC11222865; doi:10.5713/ab.23.0441)
Supplement: Supplementary file 4 [file ab-23-0441-Supplementary-Fig-1.pdf]

S Figure 1 Effect of Agaricus blazei polysaccharide on intestinal morphology of Korean quail (10×, scale bar 200 μm).

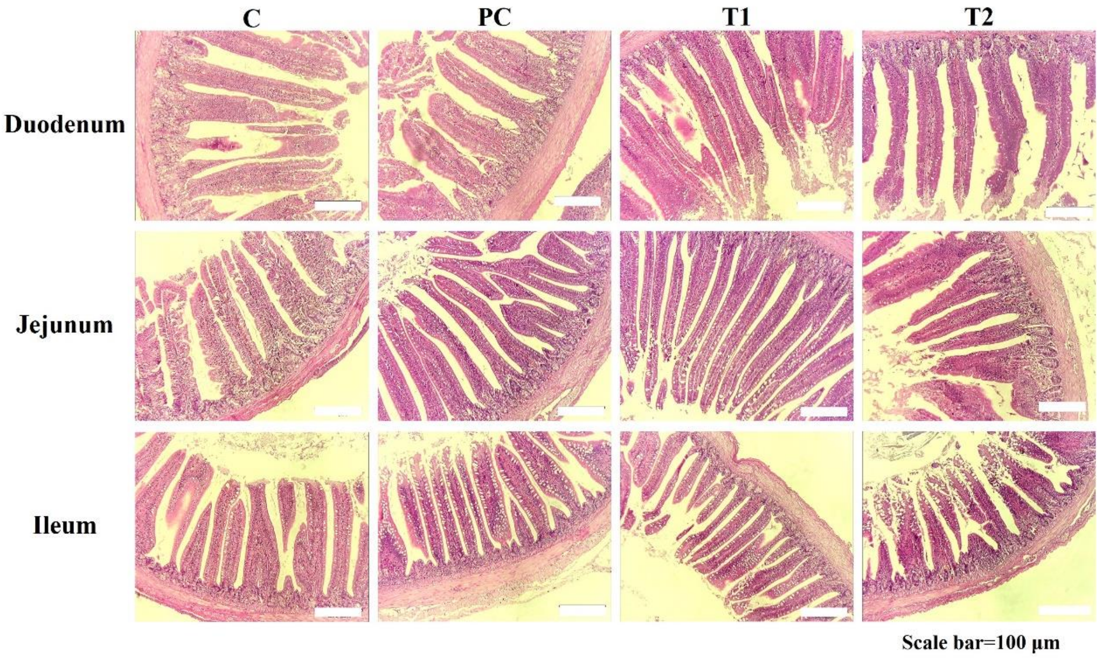

C: quails fed basal diets, T1: quails fed basal diet with 0.05% (w/w) Agaricus blazei polysaccharide, T2: quails fed basal diet with 0.1% (w/w) Agaricus blazei polysaccharide.
